# Advances in Supercapacitors Based on BiFeO_3_-Based Materials for Supercapacitor Applications

**DOI:** 10.3390/mi17070851

**Published:** 2026-07-17

**Authors:** Mohammad Aslam, Danishuddin, Mathivanan Durai, Praveen Kumar, Elangovan Erusappan, Surinder Kaur Brar, Rohit Kumar Singh Gautam, Mohd Quasim Khan

**Affiliations:** 1 School of Chemical Engineering, Yeungnam University, 280 Daehak-ro, Gyeongsan 38541, Republic of Korea; 2 Department of Biotechnology, Yeungnam University, 280 Daehak-ro, Gyeongsan 38541, Republic of Korea; 3 School of Computer Science & Artificial Intelligence, SR University, Warangal 506371, Telangana, India; 4 Department of Chemistry, Indian Institute of Technology, Indore 453552, Madhya Pradesh, India; 5 Department of Environmental Engineering, Seoul National University of Science and Technology, Seoul 01811, Republic of Korea; 6 Centre for Promotion of Research, Graphic Era (Deemed to be University), Clement Town, Dehradun 248002, Uttarakhand, India; 7 Department of Applied Sciences, School of Engineering and Technology, CGC University, Mohali 140307, Punjab, India; 8 Department of Mechanical Engineering, Teerthanker Mahaveer University, Moradabad 244001, Uttar Pradesh, India; 9 Department of Chemistry, Moradabad Muslim Degree College, Guru Jambheshwar University, Moradabad 244501, Uttar Pradesh, India

**Keywords:** BiFeO_3_, composites, supercapacitors, energy storage

## Abstract

The increasing need for electrochemical energy storage systems with high power density and long-term stability has driven intensive efforts to develop next-generation electrode materials that surpass the limitations of conventional carbonaceous and transition metal-based architectures. In this context, bismuth ferrite (BiFeO_3_; BFO), which is also known as perovskite oxide, has been employed as a promising electrode material for energy storage applications. In the past few years, BFO and its hybrid materials have emerged as promising candidates for the fabrication of supercapacitors. However, their practical development is constrained by limited intrinsic electrical conductivity, sluggish charge-transfer kinetics, and structural instability under repeated cycling. This review critically examines recent progress in BFO-based electrode materials for supercapacitor applications. The synthesis methods for BFO-based materials have been discussed, and their advantages and limitations have been compared. Furthermore, the electrochemical performance of BFO-based hybrid materials for supercapacitor application has been critically examined. The energy storage mechanism and limitations of BFO-based supercapacitors have been discussed. Future perspectives for BFO-based materials for energy storage applications have been discussed.

## 1. Introduction

The increasing global demand for energy, together with environmental concerns over fossil fuel dependence, has intensified efforts to develop efficient and sustainable energy storage technologies [1,2,3]. In particular, supercapacitors have emerged as promising electrochemical energy storage devices owing to their high power density, rapid charge–discharge kinetics, and exceptional cycling durability [4,5,6]. Nevertheless, their comparatively limited energy density relative to conventional batteries remains a major barrier to broader practical deployment, driving sustained interest in the rational design of advanced electrode materials capable of delivering enhanced electrochemical performance [7,8,9]. In this context, various electrode materials such as metal oxides [10], carbon materials [11,12,13], polymers [14], sulfides [15], perovskite materials [16], perovskite oxide-based materials [17,18,19], MXenes [20], layered double hydroxides [21], metal–organic frameworks (MOFs) [22], and covalent organic frameworks (COFs) [23] have been extensively explored as electrode materials. It should be noted that each material has its own advantages and limitations. Therefore, identifying highly efficient and stable electrode materials for the development of supercapacitors with long-term stability is of great significance.

Bismuth ferrite (BiFeO_3_; BFO) is a perovskite material, which possess decent physicochemical properties [24,25,26,27,28]. Compared with conventional carbon-based supercapacitors, which mainly rely on electric double-layer charge storage and are therefore constrained by modest energy density, BFO-based electrodes may provide additional faradaic charge storage through Fe-centered redox reactions and defect-associated electrochemical processes [29,30,31]. BFO also shows other advantages arising from its ferroelectric and dielectric properties, whereas its electrochemical response can be adjusted through composition, phase structure, doping, and interface engineering [29,30,31,32]. These features make it possible to improve charge-storage capacity and energy density beyond those typically achieved with purely carbonaceous electrodes. In addition, coupling BFO with conductive components such as carbon nanotubes or other active phases can offset its limited intrinsic conductivity and support wider operating voltage windows in asymmetric devices [33,34]. Due to the presence of efficient physicochemical properties, BFO-based materials have also been explored for other applications such as piezoelectric catalysis [35], oxygen evolution reaction (OER) [36], fuel cells [37], sensors [38], photocatalysis [39], and batteries [40]. It is also worth stating that BFO is a chemically stable electrode material and may be utilized for long-term and stable optoelectronic applications. However, its limited electrical conductivity may restrict its potential for large-scale or practical applications. Therefore, numerous strategies such as doping and forming hybrid composites, etc., were employed to enhance the electrical conductivity of BFO [41].

**Figure 1:**
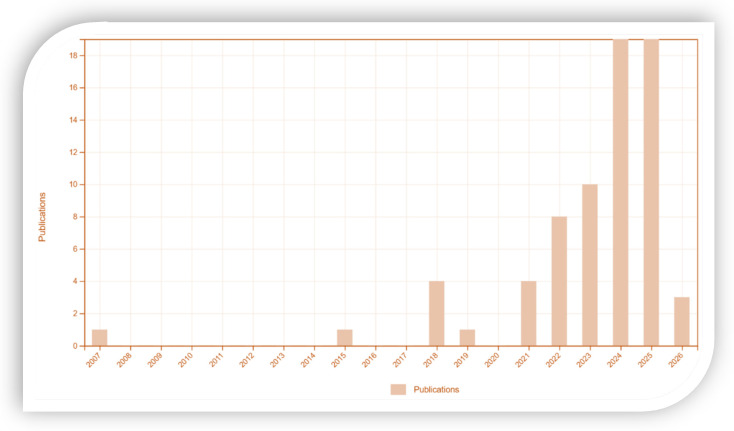
Number of publications on BFO-based supercapacitors indexed in Web of Science.

This review critically discusses the various synthesis methods for the synthesis of BFO-based materials and their applications in supercapacitors. So far, no review report has been found on BFO-based materials for supercapacitor applications. This is the first review to report on BFO-based materials for supercapacitor applications, as per the author’s knowledge and literature survey. The reported literature on BFO-based supercapacitors was searched on Web of Science, and the obtained data are illustrated in Figure 1.

## 2. Progress in the Synthesis of BFO

### 2.1. Sol–Gel Method

**Figure 2:**
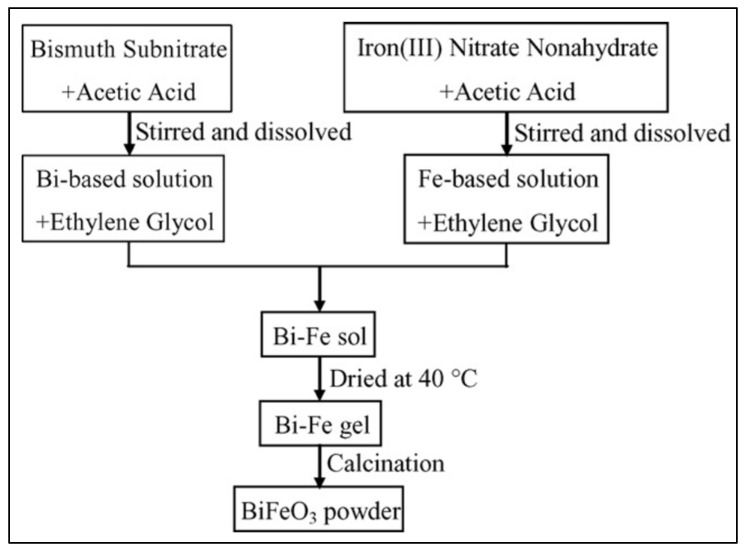
Schematic graph shows the synthesis route. Reproduced with permission [56].

Sol–gel chemistry provides substantial control over the phase evolution, morphology, and multifunctional properties of BFO, although its effectiveness depends strongly on precursor coordination and thermal treatment conditions. In the previous study [42], a modified citrate-assisted route incorporating pre-carbonization suppressed abrupt xerogel decomposition and enabled phase-pure rhombohedral BFO at a lower calcination temperature, together with nanoscale crystallites, a narrowed band gap, and improved surface area. This finding highlights an important limitation of conventional direct calcination, where uncontrolled exothermic decomposition can hinder phase selectivity. Solvent chemistry is equally pivotal: acetic acid-derived gels required thermal treatment for BFO crystallization and readily developed Bi_2_Fe_4_O_9_ impurities, whereas ethylene glycol stabilized phase-pure BFO over a broader thermal window [43]. Related studies similarly showed progressive amorphous-to-crystalline transformation with nanoscale particle formation and disruption of the cycloidal spin structure [44]. In thin films, excess Bi has been used to offset volatilization during annealing and improve low-temperature crystallization [45]. Dopant incorporation further expands the ability of sol–gel processing to tune BFO functionality. Rare-earth substitution with Gd^3+^, Ce^3+^, and La^3+^ induces lattice distortion and modifies magnetic, dielectric, and optical behavior, although phase stability remains strongly composition-dependent [46,47]. PVA-assisted processing is particularly effective in improving cation homogeneity and suppressing secondary phases, while La substitution alters dielectric and magnetic responses through controlled lattice perturbation [47]. More complex Nd^3+^/Nb^5+^ co-doping drives rhombohedral-to-mixed-phase evolution and pronounced particle size refinement, demonstrating that multisite substitution can simultaneously manipulate crystal symmetry and nanoscale dimensions [48]. More broadly, polymer-assisted routes show that metal cation-to-functional group balance is a critical but often underemphasized parameter governing phase purity at reduced processing temperatures [49]. Chelating agent selection also produces distinct structural outcomes. Tartaric acid-assisted synthesis promotes phase-pure nanoscale BFO and weak ferromagnetism associated with finite-size effects [50]. In ultrasonic spray pyrolysis, tartaric acid lowers crystallization temperature and modifies crystallite size, lattice distortion, shell porosity, and hollow-particle morphology, thereby coupling chemical complexation with magnetic response [51]. By comparison, glycine more consistently favors high-purity BFO, whereas tartaric acid and urea can promote mixed phases; nevertheless, tartaric acid-derived samples may exhibit stronger ferromagnetic behavior [52]. These contrasting observations indicate that no chelating agent is universally optimal, and phase purity must be balanced against targeted functional performance. Sol–gel processing also supports interfacial and morphology engineering beyond single-phase BFO. The incorporation of reduced graphene oxide produces BFO-based heterostructured films with improved light harvesting, charge transport, and photoelectrochemical response, although performance is highly sensitive to interfacial composition and excessive carbon loading [53]. Non-aqueous solvent effects are similarly profound: DMSO preferentially promotes Bi_2_Fe_4_O_9_ formation with anisotropic rod-like morphology, demonstrating that solvent dielectric properties, donor ability, and even solvent-derived heteroatom incorporation can redirect phase formation away from BFO [54]. Bio-template-assisted routes further enable hollow tubular BFO architectures, where intermediate thermal holding steps are critical for impurity suppression and perovskite stabilization [55]. Such architectures enhance accessible surface area and light–matter interaction, but their performance remains closely tied to annealing history and structural reproducibility. It could be concluded that the formation of BFO might be governed by a tightly coupled interplay of precursor coordination, solvent environment, polymer or chelating chemistry, dopant identity, and thermal pathway rather than calcination temperature alone. Ethylene glycol, polymer-assisted complexation, and controlled pre-carbonization generally improve low-temperature crystallization and phase purity, whereas poorly matched solvents or ligands can favor competing bismuth ferrite phases. The most effective strategies therefore move beyond simple synthesis optimization toward deliberate control of phase stability, lattice distortion, interface formation, and nanoscale morphology for application-specific magnetic, dielectric, photocatalytic, and photoelectrochemical performance [42,43,44,45,46,47,48,49,50,51,52,53,54,55,56]. The synthesis can be understood from Figure 2.

In another study, systematic variation in precursor concentration and annealing temperature allowed precise control over particle size (≈50–250 nm), impurity content, and optical band gap [57]. Photocatalytic studies indicated that particle size dominated activity when size differences were large, whereas band gap tuning and light absorption had become the controlling factors when particle sizes were comparable.

### 2.2. Hydrothermal/Solvothermal Method

**Figure 3:**
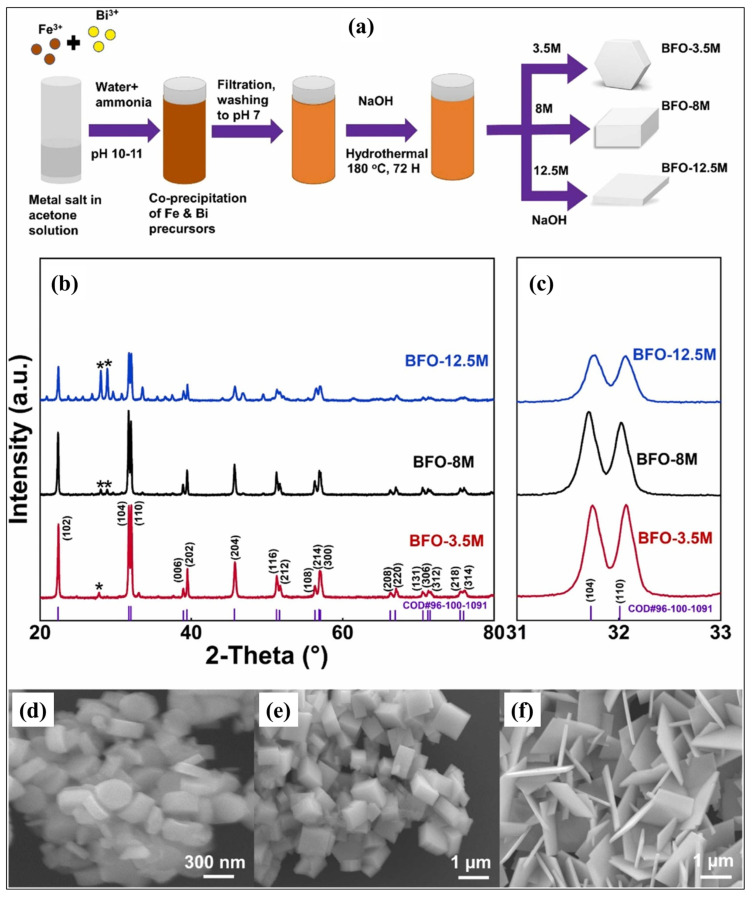
(**a**) Pictorial view of the synthesis of BFO samples. XRD results (**b**,**c**) of BFO samples. (**d**–**f**) SEM images of BFO (synthesized at different NaOH concentrations (3.5, 8 and 12.5 M)). Reproduced with permission [65].

Hydrothermal synthesis offers effective control over the phase purity, particle size, morphology, and facet exposure of BiFeO_3_ (BFO), but the outcome is highly sensitive to alkalinity, temperature, and reaction duration [58]. Systematic studies showed that phase-pure BFO forms only within an appropriate alkaline processing window, with crystallization proceeding through a dissolution–precipitation mechanism. Lower alkalinity, elevated temperature, and prolonged treatment generally promote particle growth, while the weak ferromagnetism of single-phase BFO has been linked to nanoscale effects and spin canting [58]. This strong dependence on processing conditions is a key limitation of hydrothermal routes, as small deviations can alter phase evolution and particle dimensions. Particle size reduction can markedly improve photocatalytic performance. Hydrothermally derived nano-BFO exhibited much faster methyl orange degradation than microscale BFO, with an approximately 5.5-fold higher apparent rate constant, attributed to shorter charge transport distances and reduced electron–hole recombination [59]. However, the additional annealing required in this route indicates that hydrothermal treatment alone does not always provide the desired crystallinity. Beyond particle refinement, alkaline hydrothermal processing enables three-dimensional flower-like architectures [60], oriented particles [61], and single-crystalline nanosheets or nanowires [62]. These studies collectively show that morphology can be redirected through alkalinity, temperature, reaction time, and surfactant-mediated growth, although the narrow synthesis windows may complicate reproducibility and scale-up. Hydrothermal BFO has also demonstrated functions beyond conventional photocatalysis. Nanoparticles (NPs) prepared through controlled precipitation and alkaline mineralization showed nearly complete rhodamine B degradation under repeated low-amplitude thermal cycling, demonstrating strong pyrocatalytic activity [63]. Supercritical water synthesis further offers rapid and comparatively sustainable processing, with statistical optimization identifying reaction temperature and pH as the dominant variables controlling phase purity and particle size, whereas residence time exerted a weaker influence [64]. This result is particularly important because it shifts hydrothermal optimization from empirical trial and error toward predictive process design. Facet engineering provides perhaps the clearest evidence that hydrothermal morphology control can directly govern catalytic performance [65]. The synthesis approach for the formation of BFO has been illustrated in Figure 3a. Varying NaOH concentration produced hexagonal particles, rectangular cuboids, and nanoplates with distinct exposed crystal planes, as summarized in Figure 3b–f. X-ray diffraction (XRD) confirmed phase formation, while field emission scanning electron microscopy (FESEM) revealed pronounced morphology evolution with alkalinity. Rectangular cuboids dominated by the (102) facet delivered the strongest visible-light water oxidation performance, reaching an oxygen evolution rate of 82.2 μmol h^−1^ g^−1^ without a cocatalyst, nearly twice that of the other morphologies. This enhancement was attributed to more favorable charge separation associated with facet-dependent surface energetics. It might be worth mentioning that hydrothermal synthesis is a particularly efficient approach for engineering BFO across multiple structural length scales, from nanoscale particle refinement to anisotropic growth and facet-selective architectures. Nevertheless, its major weakness is the strong coupling between alkalinity, temperature, pressure, and residence time, which can narrow the processing window for phase-pure products. The most convincing studies therefore move beyond simple particle size control and demonstrate direct links between hydrothermal parameters, exposed facets, charge separation, and catalytic or pyrocatalytic performance [58,59,60,61,62,63,64,65].

Hydrothermal and solvothermal strategies provide broad control over BFO facet exposure, dopant incorporation, phase selectivity, and hierarchical morphology. A one-pot polyethylene-glycol-assisted route produced phase-pure pill- and rod-like BFO dominated by exposed {111}(c) facets, which showed stronger visible-light response than {100}(c)-terminated cubes, reinforcing the importance of surface crystallography in governing optoelectronic behavior [66]. Similarly, a combined sol–gel/hydrothermal route enabled La-doped BFO microspheres and submicron tiles, with morphology strongly dependent on alkalinity and crystallization occurring at markedly lower temperature than conventional sol–gel processing [67]. These studies show that hybrid processing can reduce thermal demand while preserving morphological control, although strong sensitivity to mineralizer concentration remains a practical limitation. Hydrothermal doping further demonstrates how local lattice distortion can be translated into multifunctional response. Sb substitution at Fe sites reduced rhombohedral distortion toward a more ideal perovskite structure [68], whereas Yb incorporation retained the rhombohedral framework while refining particle size and enhancing magnetization through lattice distortion and partial disruption of the cycloidal spin structure [69]. Such results indicate that dopant effects cannot be separated from nanoscale size evolution, and improvements in magnetism should therefore not be attributed solely to substitution chemistry. Controlled precipitation under solvothermal conditions offers another route to phase-pure BFO. HMTA-mediated synthesis enabled rapid crystallization, with precipitant concentration governing both phase formation and morphology; optimized samples showed improved dielectric, magnetic, ferroelectric, and optical responses [70]. More broadly, hydrothermal phase selection is highly sensitive to Bi/Fe stoichiometry, solution chemistry, and temperature, allowing deliberate switching between perovskite BFO and sillenite-type Bi_25_FeO_40_ [71]. This phase competition is important because it illustrates that nominal precursor composition alone does not determine the final bismuth ferrite structure. Morphology engineering has also advanced from compact particles to hollow and anisotropic architectures. Hollow BFO spheres were obtained through coordinated control of complexation, precipitation, and hydrothermal growth, although prolonged treatment or excess complexing agent promoted Bi_2_Fe_4_O_9_ and Bi_2_O_3_ impurities [72]. The need for post-synthetic acid washing further highlights the narrow phase purity window of such hierarchical routes. Microwave-assisted hydrothermal processing, in contrast, enabled rapid evolution from microspheres to microcubes through facet-selective adsorption of PMVEMA, demonstrating how polymer additives can redirect anisotropic growth under accelerated heating [73]. Low-alkalinity hydrothermal synthesis also produced single-phase submicron BFO with weak room-temperature ferromagnetism, attributed to size-induced disruption of the bulk spiral spin structure [74]. Finally, the above-mentioned studies reveal that advanced hydrothermal processing was most effective when reaction chemistry was deliberately coupled with facet-selective additives, dopant-induced lattice distortion, and controlled nucleation. However, the same chemical sensitivity that enables precise structural tuning also creates narrow processing windows, phase competition, and reproducibility challenges. Future progress should therefore prioritize a mechanistic understanding of additive–facet interactions and quantitative links between hydrothermal conditions, lattice distortion, exposed surfaces, and functional performance rather than relying mainly on empirical morphology optimization [66,67,68,69,70,71,72,73,74].

### 2.3. Sonochemical/Precipitation Method

**Figure 4:**
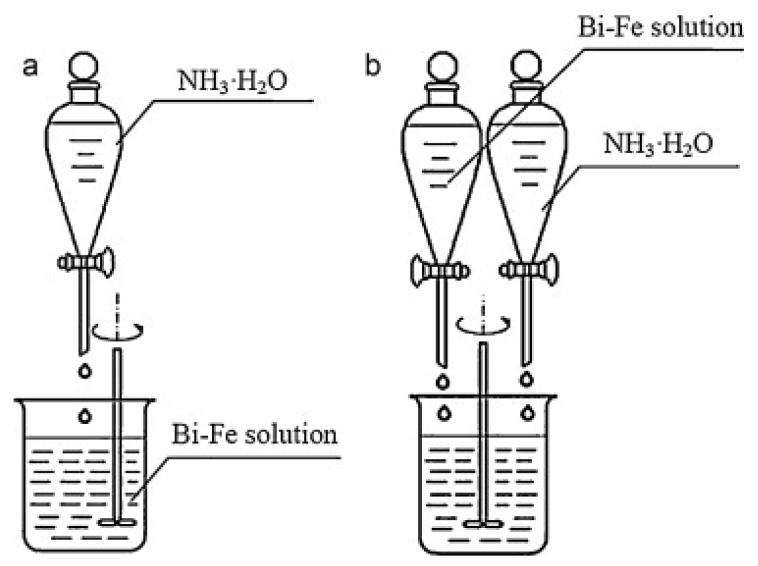
Schematic graph shows (**a**) traditional co-precipitation and (**b**) homogenous co-precipitation methods. Reproduced with permission [76].

Sonochemical processing has emerged as an effective route for enhancing the multiferroic response of doped BFO. Sm^3+^/Zr^4+^ co-substitution improved magnetic, ferroelectric, and magnetodielectric behavior through combined lattice distortion, particle size refinement, and reduced leakage current [75]. Sm^3+^ substitution was particularly effective in strengthening magnetization and polarization, while additional Zr^4+^-induced strain further promoted multiferroic coupling. However, because dopant incorporation and particle size reduction occurred concurrently, their individual contributions to the enhanced response remain difficult to decouple. Chemical co-precipitation offers a lower-complexity alternative for phase-pure BFO, but its success depends critically on precipitation homogeneity, pH control, and Bi stoichiometry. Synchronized reagent addition under constant pH promoted uniform nucleation and rhombohedral BFO formation, while slight Bi excess suppressed competing phases (Figure 4) [76].

Related work confirmed that an optimized alkaline window improved phase purity, dielectric behavior, and leakage characteristics [77]. Co-precipitated amorphous precursors also enabled BFO crystallization at comparatively moderate temperatures, with increasing calcination enhancing crystallinity, although grain growth accompanied these improvements [78]. Thus, thermal treatment presents an inherent trade-off between structural ordering and nanoscale retention. Precipitant chemistry provides an additional method of controlling phase evolution. Mixed ammonia/ammonium bicarbonate precipitation suppressed secondary phases and produced visible-light-active BFO with strong methyl orange degradation performance [79]. Rare-earth-modified BFO prepared through co-precipitation showed tunable optical and photoelectrochemical behavior, although dopant incorporation also introduced partial loss of crystallinity [80], indicating that compositional modification might improve electronic response at the expense of structural order. A direct comparison of normal and reverse co-precipitation further demonstrated that reagent addition sequence can determine whether phase-pure BFO or impurity-rich products are obtained [81]. The finer particles produced under selected conditions also exhibited weak ferromagnetism, consistent with disruption of the long-period cycloidal spin structure and increased uncompensated surface spins. Alternative carbonate-mediated precipitation reduced the temperature required for BFO formation by generating highly reactive oxide intermediates during precursor decomposition [82]. This route suppressed persistent secondary phases more effectively than conventional solid-state processing and retained nanoscale features associated with weak ferromagnetism. Notably, these studies suggested that co-precipitation is not merely governed by nominal pH or calcination temperature; precursor speciation, addition sequence, precipitant identity, and Bi volatility collectively determine phase purity and functional performance. Its main advantages are chemical simplicity and relatively low processing temperature, whereas sensitivity to local pH gradients, post-calcination grain growth, and recurring impurity formation remain key limitations [76,77,78,79,80,81,82].

### 2.4. Microwave Method

Microwave-assisted processing offers a rapid alternative to conventional BFO synthesis by combining volumetric heating with accelerated nucleation and crystallization. Phase-pure BFO NPs were obtained within minutes, with phase evolution strongly dependent on irradiation time [83]. The reported periodic appearance of single-phase BFO was attributed to rapid thermal cycling and repeated decomposition–recrystallization, indicating that microwave processing could shorten synthesis substantially but might also have introduced a narrow time-dependent phase window. Microwave-assisted hydrothermal synthesis with CTAB further produced phase-pure nanoscale BFO with abundant surface hydroxyl groups and enhanced visible-light photocatalytic activity, reaching nearly fivefold higher Rhodamine B degradation than bulk BFO [84]. The improvement was associated with more efficient charge separation and reactive hole- and hydroxyl radical-mediated pathways, although acid washing and wet grinding remained necessary to achieve the final purity and particle characteristics. Microwave routes also enable morphology-controlled BFO without prolonged thermal treatment. Microcubes prepared using a microwave-assisted hydrothermal Pechini process retained structural stability without post-calcination and exhibited magnetic relaxation dominated by ferromagnetic domain-wall dynamics rather than spin-glass behavior [85]. Likewise, nanoscale BFO cubes maintained switchable ferroelectric polarization after deposition on conductive oxide substrates, supporting their potential for miniaturized multiferroic devices [86]. These studies demonstrate that the key advantage of microwave processing lies not only in faster synthesis but also in preserving functional ferroic behavior at reduced particle dimensions. Microwave-assisted approaches have additionally been combined with chemical substitution and thin-film processing to tune electromagnetic properties. Nd/Cr-modified BFO showed dopant-dependent dielectric and magnetic responses linked to lattice distortion, modified super-exchange interactions, and enhanced dipolar polarization, with Cr substitution improving dielectric loss characteristics relevant to microwave absorption [87]. However, because these compositions were produced through sol–gel auto-combustion rather than direct microwave synthesis, their property enhancement should be distinguished from genuine microwave processing effects. In thin films, microwave power strongly influenced phase evolution and magnetic ordering, with an intermediate irradiation window yielding phase-pure BFO and pronounced ferromagnetism, whereas insufficient or excessive power produced paramagnetic or mixed-phase states [88]. Notably, microwave-assisted synthesis provides major advantages in reaction speed, reduced thermal exposure, and morphology control, but phase purity remains highly sensitive to irradiation time, power, alkalinity, and post-treatment. The strongest evidence therefore supports microwave processing as a kinetic tool for controlling nucleation and functional nanostructure formation rather than as a universally phase-selective route [83,84,85,86,87,88].

### 2.5. Solid-State Method

Mechano-thermal and high-energy ball milling routes provide scalable pathways for BFO synthesis and microstructural refinement, but phase evolution is strongly dependent on milling energy and subsequent thermal treatment. Mechanical activation of Bi_2_O_3_/Fe_2_O_3_ mixtures lowers the temperature required for BFO formation by increasing defect density and interfacial reactivity; however, milling alone does not necessarily yield phase-pure BFO [89]. An optimum energy window minimizes secondary phases, whereas excessive milling promotes contamination from the milling media. This trade-off between enhanced reactivity and mechanically induced impurities is a central limitation of high-energy processing. Ball milling has also been coupled with thin-film fabrication, where nanosized precursors obtained by intensive milling support the formation of rhombohedral BFO after spray deposition and annealing [90]. Nevertheless, the requirement for high-temperature post-treatment reduces part of the energetic advantage expected from mechanical activation. Previously, BFO-based materials have been prepared with good phase purity [91,92,93,94,95]. Mechanochemical processing has enabled the direct room-temperature formation of nanoscale perovskite BFO with distinctive core–shell features, demonstrating that sufficiently intense mechanical treatment can, under selected conditions, drive solid-state reaction without conventional calcination [94]. The contrasting outcomes of these studies indicate that mechanochemical efficiency depends not only on milling intensity but also on precursor state, reactor configuration, and contamination control. Mechanical processing is particularly effective for constructing BFO-based heterostructures and composites. The mechano-thermal synthesis of BFO-Fe_2_O_3_ generated intimate interfacial contact and improved photocatalytic activity through reduced charge recombination, although the product remained deliberately multiphase rather than phase-pure BFO [91]. Similarly, carbonyl iron/BFO composites showed enhanced microwave absorption through interfacial polarization and improved impedance matching [93]. High-energy milling of BLFO-BaFe_12_O_19_ composites reduced interphase separation and strengthened magnetic exchange interactions compared with simple solid-state mixing [96]. These results underscore that the main value of ball milling often lies in interface engineering rather than BFO crystallization alone. Advanced combinations of milling and rapid densification further expand property control. La-substituted BFO processed through high-energy milling, cryomilling, rapid thermal treatment, and spark-plasma sintering retained refined crystallites and elevated micro-strain, leading to enhanced magnetization [95]. Mechanical alloying of BFO-based morphotropic solid solutions likewise enabled particle size-dependent phase evolution and improved dielectric and ferroic behavior [97]. However, because such routes combine several severe-processing steps, attributing property enhancement to a single variable remains difficult. Some reported mechano-thermal examples fall outside direct BFO synthesis and should be treated cautiously. The BaTiO_3_–CoFe_2_O_4_ system demonstrates the broader utility of mechanically activated processing for multiferroic composites, but it does not provide direct evidence for BFO phase formation [92]. Overall, mechanochemical methods are attractive for scalable particle refinement, defect generation, and interface construction, yet their principal limitations include media contamination, incomplete phase conversion, high post-annealing demand, and difficulty in separating milling-induced strain effects from compositional or size contributions [89,90,91,92,93,94,95,96,97]. The synthesis and related parameters for BFO-based materials have been summarized in Table 1.

## 3. Electrochemical Energy Storage

### 3.1. BFO-Based Materials

**Figure 5:**
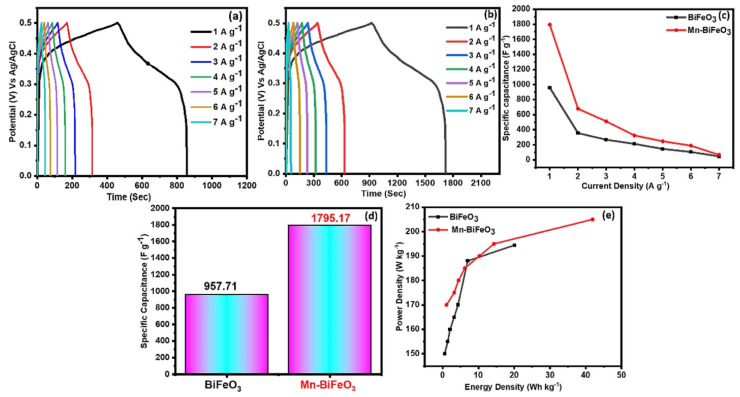
GCD characteristics of (**a**) pristine BFO and (**b**) Mn-doped BFO-based electrodes at different current densities (1–7 A g^−1^); (**c**) comparison of specific capacitance as a function of current density for pristine and Mn-doped BFO. (**d**) Comparison of the maximum specific capacitance, showing enhancement from 957.71 F g^−1^ for BFO to 1795.17 F g^−1^ for Mn-BFO at 1 A g^−1^; and (**e**) Ragone-type energy–power density relationship. Reproduced with permission [99].

Perovskite, or BFO, is a promising electrode material for supercapacitors owing to its high theoretical capacity and wide operating voltage arising from reversible multivalent cation redox reactions. However, its practical performance is often limited by sluggish charge-transfer kinetics and poor cycling stability associated with its dense crystal structure. Defect and structural engineering strategies have been shown to effectively overcome these limitations. In a previous study, Agrawal et al. [30] tuned Bi stoichiometry in Bi_1+x_FeO_3_ nanocrystals and observed that Bi_1.05_FeO_3_ was the optimum composition, with the lowest secondary-phase content (4.1%), reduced band gap, and improved oxygen vacancy/electronic structure characteristics. The optimized electrode delivered 222.2 F g^−1^ in 1 M KOH, while the Bi_1.05_FeO_3_//activated carbon asymmetric device achieved 124.9 Wh kg^−1^ at 1750 W kg^−1^ and retained 88% capacitance after 10,000 cycles. This work convincingly links Bi stoichiometry and phase purity with electrochemical performance using XRD and XAS, although the exact contribution of oxygen vacancies and residual Bi_25_FeO_40_ to charge storage remains partly inferential. Venkat et al. [31] prepared 2 mol% Nd-doped BFO NPs through microwave-assisted sol–gel combustion, with Nd substitution reducing crystallite size from ~62 to ~18 nm and improving pseudocapacitive redox activity in 3 M KOH. Nd-BFO delivered 24 F g^−1^ at 2 A g^−1^, an energy density of 1.5 Wh kg^−1^, and 80% capacitance retention after 2000 cycles, together with lower charge-transfer resistance than pristine BFO. In a report, V-doped BFO synthesized via electrochemical deposition followed by electrochemical induction exhibited a stable nanoflake network with a high concentration of oxygen vacancies, leading to enhanced Li^+^ diffusion, a superior rate capability, and improved structural stability [98]. First-principles calculations confirmed that V doping and oxygen vacancies strengthen the built-in electric field and create fast ion-transport pathways. Flexible asymmetric micro-supercapacitors based on this electrode delivered a high areal energy density of 7.33 µWh cm^−2^. In another work, hydrothermally synthesized Mn-doped BFO was also employed for energy storage applications. The galvanostatic charge–discharge (GCD) curves of the BFO and Mn-BFO have been displayed in Figure 5a and Figure 5b, respectively. The relationship between BFO and Mn-BFO and the specific capacitance of Mn-BFO are shown in Figure 5c and Figure 5d, respectively, whereas the power density-versus-energy density relationship of BFO and Mn-BFO is shown in Figure 5e. Mn-BFO exhibited a high surface area (49 m^2^ g^−1^), low charge-transfer resistance, and an exceptionally high specific capacitance of 1795 F g^−1^ at 1 A g^−1^ in alkaline electrolyte, highlighting the role of Mn incorporation in improving electrical conductivity and interfacial charge transfer [99].

**Figure 6:**
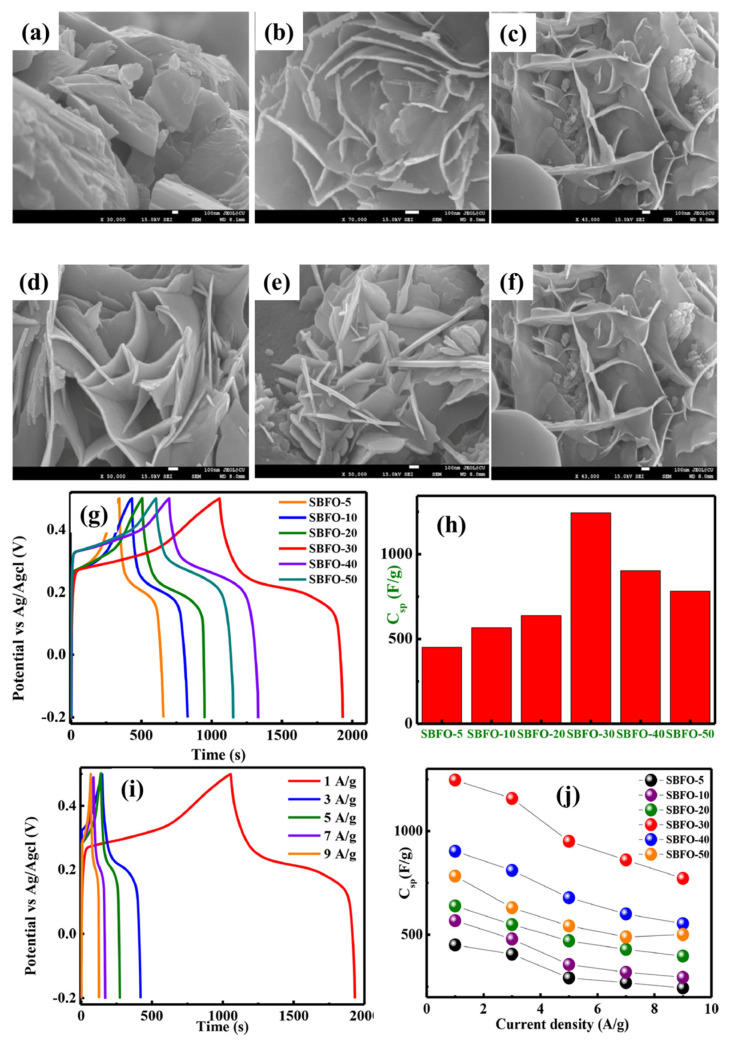
FESEM images of the prepared (**a**) SBFO-5, (**b**) SBFO-10, (**c**) SBFO-20, (**d**) SBFO-20, (**e**) SBFO-40 and (**f**) SBFO-50. GCD curves of (**g**) Sr_x_Bi_1−x_FeO_3_ (0.05 ≤ *x* ≤ 0.5) electrodes at 1 A g^−1^ and (**h**) bar graph for the obtained specific capacitance. (**i**) GCD curves of SBFO-30-based electrode at different current densities (1–9 A g^−1^). (**j**) Specific capacitance versus current density data for different electrodes. Reproduced with permission [101].

Li et al. [100] developed a pH-decoupled aqueous AC//BFO hybrid supercapacitor in which the BFO anode operates in 2 M LiOH and the activated carbon cathode operates in 1 M Li_2_SO_4_, with the two electrolyte environments separated by a tailored low-impedance cation-exchange membrane. Two interpenetrating network membranes were compared, and the sulfonic acid-rich CEM-2 exhibited higher water uptake, a smaller thickness of 40 μm, and lower membrane resistance of 3.5 Ω·cm^2^ than CEM-1, thereby facilitating faster cation transport. The BFO electrode displayed mixed diffusion- and capacitance-controlled charge storage associated with reversible Bi/Fe redox chemistry and oxygen vacancy-assisted anion intercalation pseudocapacitance, while the activated carbon cathode contributed predominantly to electric double-layer storage. By spatially separating alkaline and neutral electrolytes, the pH-decoupling configuration suppressed parasitic water decomposition and extended the stable device voltage to approximately 2.2 V. Consequently, the CEM-2-based device delivered 61 F g^−1^ at 1 A g^−1^, coulombic efficiencies of 97–103%, a comparatively low series resistance of 39.74 Ω, and a maximum energy density of 41.0 Wh kg^−1^ at 1.1 kW kg^−1^. The device retained 73.3% of its capacitance after 5000 cycles at 3 A g^−1^. This study was particularly significant because it shifted performance optimization beyond electrode composition alone, demonstrating that membrane chemistry, ion-transport resistance, and local electrolyte pH could be co-engineered to regulate interfacial redox processes and widen the aqueous electrochemical stability window. Nevertheless, the moderate long-term capacitance retention and pronounced self-discharge from 2.2 to 0.51 V over 24 h indicated that membrane durability, crossover control, and parasitic interfacial reactions remained important barriers to practical implementation. Tripathi et al. [101] developed hydrothermally synthesized Sr_x_Bi_1−x_FeO_3_ nanostructures and demonstrated that controlled Sr^2+^ substitution could significantly enhance the pseudocapacitive performance of BFO. Among the investigated compositions, SBFO-30 showed the most favorable combination of structural modification, porosity, and ion-transport kinetics, delivering approximately 1200 F g^−1^ at 1 A g^−1^ with 89% retention after 5000 cycles. As shown in Figure 6a–f, the electrodes consist of interconnected flower-like nanoflake assemblies with widths of about 15–35 nm, creating open pathways for electrolyte penetration and increasing the availability of redox-active sites. The comparatively well-developed porous architecture of SBFO-30 is consistent with its highest BET surface area and pore size, although its superior response also reflects favorable phase evolution and enhanced diffusion kinetics. The GCD profiles in Figure 6g–j are distinctly non-linear, confirming Faradaic pseudocapacitive storage rather than ideal electric double-layer behavior. SBFO-30 exhibits the longest discharge time, the highest specific capacitance, and a relatively small IR drop, indicating improved charge storage and reduced interfacial resistance. Its capacitance decreases with increasing current density because rapid polarization limits electrolyte access to internal active sites, yet it remains superior to the other Sr-doped compositions over the tested range.

**Figure 7:**
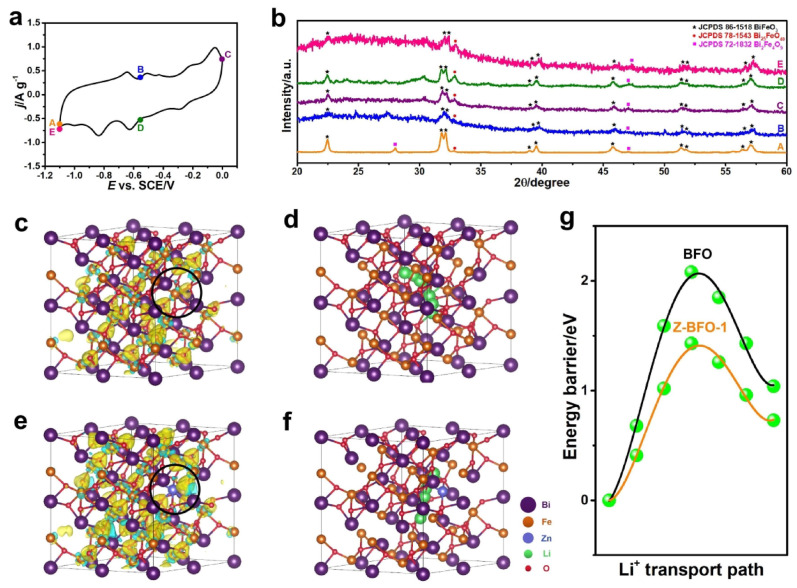
Proposed charge-storage and Li^+^-transport mechanism of the Zn-doped BFO (Z-BFO-1) electrode: (**a**) CV profile showing the selected charge/discharge states A–E; (**b**) corresponding ex situ XRD patterns revealing retention of the principal BFO framework during cycling; differential charge-density distributions and calculated Li^+^ migration pathways for (**c**,**d**) pristine BFO and (**e**,**f**) Z-BFO-1; and (**g**) calculated Li^+^ migration-energy profiles, demonstrating a reduction in the migration barrier from 2.08 eV for BFO to 1.43 eV for Z-BFO-1. Zn^2+^ substitution and associated oxygen vacancy formation enhance local charge redistribution and built-in electric fields, thereby facilitating Li^+^ transport and pseudocapacitive charge storage. Reproduced with permission [102].

Similarly, Yu et al. [102] developed a Zn-doped BFO anode, BiFe_0.95_Zn_0.05_O_3_-δ (Z-BFO-1), to improve the intrinsically sluggish ion transport of BFO for flexible battery–supercapacitor hybrid devices. Moderate Zn^2+^ substitution at Fe^3+^ sites increased the oxygen vacancy concentration while largely preserving the rhombohedral BFO framework, whereas higher Zn loading promoted impurity phases and inferior electrochemical performance. Z-BFO-1 delivered a specific capacitance of 223 F g^−1^ at 0.2 A g^−1^, and its charge-storage mechanism is illustrated in Figure 7. The CV curve in Figure 7a identifies states A–E selected for ex situ structural analysis, while the corresponding XRD patterns in Figure 7b show that the principal BFO reflections persist throughout charging and discharging, supporting largely reversible pseudocapacitive storage without catastrophic collapse of the host lattice, although Bi_25_FeO_40_ and Bi_2_Fe_4_O_9_ impurity reflections remain detectable. DFT-derived differential charge-density maps further show that pristine BFO (Figure 7c) has comparatively weaker local charge redistribution, whereas Z-BFO-1 (Figure 7e) exhibits more pronounced charge-accumulation and charge-depletion regions. This redistribution is attributed to the aliovalent Zn^2+^-for-Fe^3+^ substitution and associated charge compensation, including oxygen vacancy formation, which strengthens local built-in electric fields. The calculated Li^+^ migration pathways around Fe in BFO (Figure 7d) and around Zn in Z-BFO-1 (Figure 7f) consequently yield markedly different transport barriers; as summarized in Figure 7g, Zn doping lowers the calculated Li^+^ migration barrier from 2.08 to 1.43 eV. Thus, the enhanced pseudocapacitance is best interpreted as a coupled defect engineering effect in which Zn-induced oxygen vacancies provide more favorable ion-transport space and the modified local electronic field facilitates Li^+^ migration. Critically, however, the mechanistic claim should be viewed as strongly supported rather than fully proven because the direct link between the calculated migration pathway and the experimentally operating intercalation pathway is inferential, and persistent secondary phases complicate the exclusive attribution of charge storage to the Z-BFO-1 host lattice.

Bhat et al. [103] investigated Nd/Co co-doped BFO NPs, Bi_1−x_Nd_x_Fe_1−y_Co_γ_O_3_ (x = y = 0.05–0.20), prepared through high-energy mechanical milling followed by sintering, as multifunctional dielectric and supercapacitor materials. Increasing Nd/Co substitution progressively reduced grain size and induced a rhombohedral-to-mixed rhombohedral/tetragonal and ultimately tetragonal structural evolution. Electrochemically, the redox-active CV profiles indicated predominantly pseudocapacitive/Faradaic charge storage, attributed mainly to Bi^3+^/Bi^0^ redox chemistry, with the highly doped BNFCO-0.20 composition showing the best performance: 510 F g^−1^ from CV analysis and 466 F g^−1^ from GCD at 20 mA, together with low solution and charge-transfer resistances of 0.43 and 1.99 Ω, respectively. The improvement was associated with dopant-induced structural distortion, smaller grains, enhanced charge transport, and electronic structure modification. However, the mechanistic interpretation remains largely correlative because direct operando evidence for the proposed redox pathway and the individual roles of Nd and Co is absent; moreover, the study relies on a three-electrode configuration, limiting direct assessment of practical device-level energy and power performance. Ganie et al. [104] synthesized Yb^3+^/Cr^3+^ co-doped BFO nanostructures using a hydrothermal route and showed that co-doping preserved the rhombohedral BFO phase while reducing crystallite and grain size and narrowing the band gap from 2.23 to 2.12 eV. The most highly doped Bi_0.9_Yb_0.1_Fe_0.95_Cr_0.05_O_3_ electrode exhibited the best electrochemical response, delivering 596 F g^−1^ from CV and 495 F g^−1^ at 10 mA cm^−2^ from GCD with 91% retention after 10,000 cycles. The enhancement was attributed to finer grains, increased surface/space-charge contribution, improved conductivity, and facilitated Faradaic redox processes. However, the proposed mechanism remains largely indirect because the study does not provide operando identification of the active redox species, and the three-electrode evaluation limits the direct assessment of practical device performance. These results underline the effectiveness of dopant-induced defect engineering and nanostructure control in overcoming the ion-transport limitations of BFO, positioning doped BFO systems as viable candidates for high-performance supercapacitors and hybrid energy storage devices.

### 3.2. BFO/Metal Oxide-Based Materials

**Figure 8:**
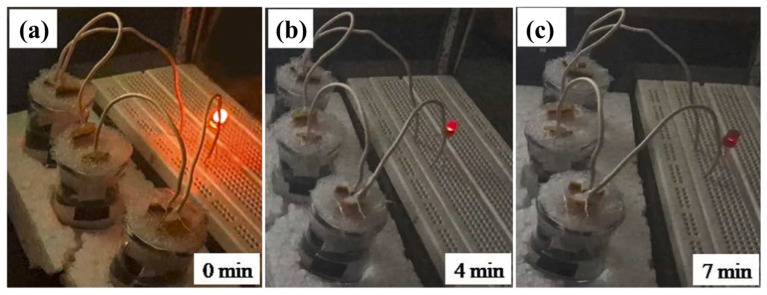
(**a**–**c**) Red LED (1.8 V) powered via MO35//MO35 cells. Reproduced with permission [107].

Recent studies highlight the effectiveness of heterojunction and composite engineering in enhancing the energy storage and conversion performance of BFO-based materials [105]. A PVDF-supported β-Bi_2_O_3_/BFO p-n heterojunction was synthesized via a hydrothermal route. The PVDF-β-Bi_2_O_3_/ BFO electrode also demonstrated superior capacitive behavior, delivering a specific capacitance of 300 F g^−1^ at 1 A g^−1^ in 3 M KOH, along with a symmetric device energy density of 10.08 Wh kg^−1^, power density of 999 W kg^−1^, and 88.7% capacitance retention over 10,000 cycles. In another study [106], mesoporous Bi_2_O_3_/BFO rectangular nanorods were derived from Prussian blue analogs exhibiting high specific capacitance (741 F g^−1^) and excellent cycling stability, benefiting from their porous architecture that mitigates structural degradation during repeated charge–discharge processes. When paired with NiCo-LDH/rGO as the positive electrode, asymmetric devices delivered an energy density of 59.7 Wh kg^−1^ at 800 W kg^−1^ with stable capacity retention. Similarly, MoO_3_@BFO nanocomposites exhibited markedly improved charge-storage properties, with the optimized composite containing 35 wt % MoO_3_ showing the best performance [107]. Structural analysis confirmed the formation of a Bi_2_Mo_3_O_12_ phase and increased oxygen vacancy concentration, while the interlinked BFO NPs and MoO_3_ nanobelts facilitated efficient charge transport. As a result, the composite delivered a high specific capacitance of 515 F g^−1^ over a wide potential window (−0.4 to 1 V) and exceptional cycling stability with 118% capacitance retention after 20,000 cycles. The red LED powered by MO35//MO35 cells is displayed in Figure 8a–c.

Recycled polyethylene (PE) substrates derived from toothpaste tubes have been successfully employed to fabricate flexible supercapacitors by printing conductive graphene inks and incorporating BFO/BaTiO_3_ (BFO/BT) ceramic additives [108]. The introduction of BFO/BT markedly enhanced electrochemical performance, increasing the specific capacitance and energy density from 312.4 F g^−1^ and 62.4 Wh kg^−1^ to 721.8 F g^−1^ and 144.6 Wh kg^−1^, respectively, due to the presence of multiple redox-active centers and oxygen vacancies. The replacement of the conventional PVA/H_3_PO_4_ gel electrolyte with sea water yielded a slightly lower capacitance (563.7 F g^−1^) but maintained competitive energy density while eliminating environmental toxicity, highlighting the feasibility of fully eco-friendly supercapacitor designs. Collectively, these studies underscore the promise of BFO-based heterostructures in enabling high-energy, durable, and sustainable supercapacitors for next-generation wearable and portable energy storage applications.

### 3.3. BFO/Carbon-Based Materials

**Figure 9:**
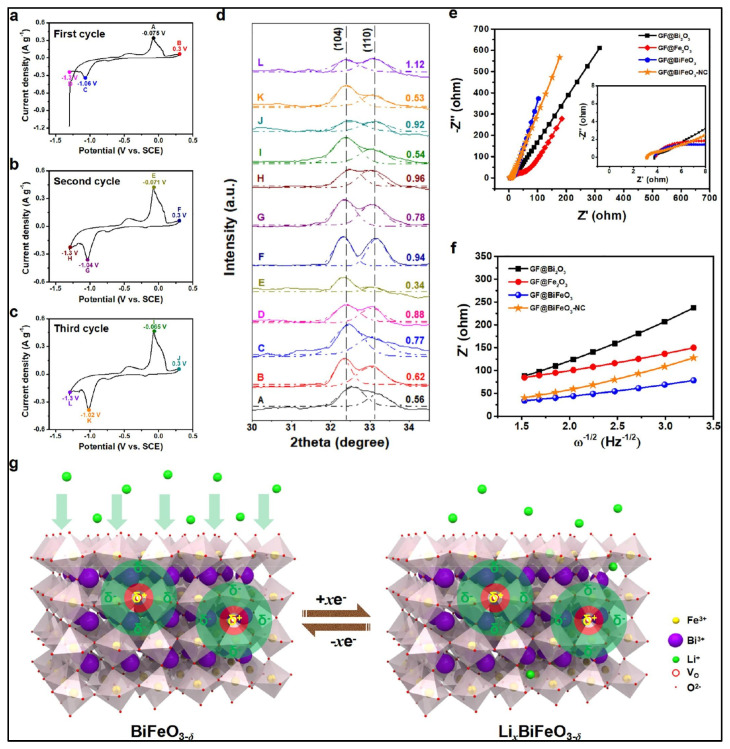
Proposed charge-storage mechanism examinations of GF@BFO-based electrode: (**a**–**c**) First, second, and third CV cycles showing reproducible electrochemical redox behavior. (**d**) Ex situ XRD patterns acquired at various potentials A–L. (**e**) Nyquist plots of the different electrodes, with an enlarged view of the high-frequency region. (**f**) Z′ versus ω^−1/2^ plots. (**g**) Schematic illustration of charge-storage mechanism [109].

Pan et al. [109] developed an oxygen-deficient BFO nanoflake anode stabilized with an N-doped carbon layer (BFO-NC) for flexible battery–supercapacitor hybrid devices. BFO nanoflakes were electrodeposited onto graphite fibers and subsequently carbonized in the presence of melamine, which introduced both an N-doped carbon coating and oxygen vacancies. Electrochemical measurements showed that the BFO-NC electrode delivered a high specific capacity of 190 mAh g^−1^ at 1 A g^−1^, together with improved rate capability and cycling stability compared with pristine BFO. The charge-storage mechanism of the oxygen-deficient BFO-based electrode was investigated through cyclic voltammetry, ex situ X-ray diffraction, and electrochemical impedance analysis. The first three CV cycles in Figure 9a–c show reproducible redox behavior, indicating a largely reversible electrochemical process. Ex situ XRD patterns collected at selected potentials A–L (Figure 9d) reveal reversible variations in the relative intensities and positions of the characteristic (104) and (110) reflections of rhombohedral BFO. Importantly, no characteristic diffraction peaks of metallic Bi were detected after repeated cycling, suggesting that charge storage does not proceed through the irreversible BFO decomposition previously associated with Bi^3+^ extraction in strongly alkaline media. Instead, the potential-dependent structural changes support reversible Li^+^ insertion and extraction accompanied by lattice distortion, which can be represented as BFO + xLi^+^ + xe^−^ ↔ Li_x_BFO. The Nyquist plots in Figure 9e further compare the interfacial and transport behavior of the electrodes, while the linear dependence of Z′ on ω^−1/2^ in Figure 9f provides information on Li^+^ diffusion kinetics. The mechanism proposed in Figure 9g therefore links energy storage to reversible Li^+^ intercalation/de-intercalation within the oxygen-deficient BFO lattice. In this model, oxygen vacancies create positively polarized local regions and associated internal electric fields that can promote ion migration through the FeO_6_-based framework, thereby contributing to the intercalation pseudocapacitive response, rate performance, and cycling stability of the electrode.

**Figure 10:**
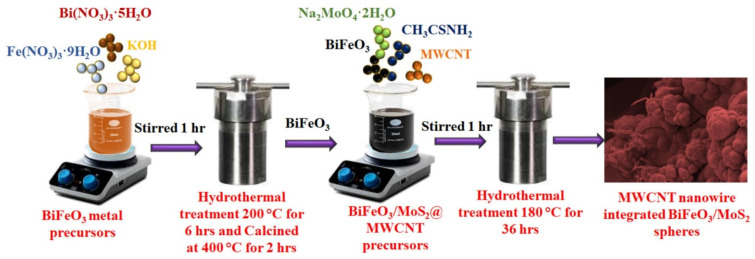
Schematic graph illustrating the formation of BFO-based composite. Reproduced with permission [113].

Pecenek et al. [110] reported a high-energy asymmetric hybrid supercapacitor by integrating graphitic carbon nitride-modified BFO (BFO/g-C_3_N_4_) as the positive battery-type electrode with orange peel-derived activated carbon as the negative electrode. BFO was prepared hydrothermally, whereas g-C_3_N_4_ was obtained through the thermal treatment of urea and subsequently coupled with BFO through ball milling. The incorporation of two-dimensional g-C_3_N_4_ markedly altered the morphology of BFO and improved its electrochemical response, which the authors attributed to enhanced electronic transport and synergistic interaction between the two components. The BFO/g-C_3_N_4_ electrode displayed pronounced Bi^3+^/Bi^2+^ and Fe^3+^/Fe^2+^ redox activity and a predominantly diffusion-controlled battery-type charge-storage mechanism, with a reported b-value close to 0.5. When assembled with biomass-derived activated carbon, the asymmetric device delivered a high specific capacitance of 330 F g^−1^ at 1 A g^−1^, operated across a wide voltage window of approximately 2.4 V, and achieved a maximum energy density of 244.8 Wh kg^−1^ at a power density of 1.155 kW kg^−1^. The device also maintained 60% capacity after 5000 cycles at 20 A g^−1^. The charge-storage mechanism of BFO is strongly dependent on electrode polarity and electrolyte environment. When BFO operates as an anode, negative polarization favors reduction-coupled ion insertion into the perovskite framework. For oxygen-deficient BFO, ex situ XRD evidence supports reversible Li^+^ insertion/extraction accompanied by lattice distortion rather than irreversible decomposition to metallic Bi. Oxygen vacancies provide additional ion-accessible pathways and generate local electric fields that facilitate Li^+^ migration through the FeO_6_-based framework, thereby producing a mixed intercalation pseudocapacitive and diffusion-controlled response [109]. Related BFO anodes also show coupled capacitive and diffusion contributions, with oxygen vacancies promoting ion transport and, in alkaline media, anion-associated intercalation pseudocapacitance [100]. When BFO functions as a cathodic or positive electrode, the mechanism differs because charge storage proceeds within a more positive potential range and is dominated by reversible Faradaic redox transitions rather than reduction-driven Li^+^ insertion. In the BFO/g-C_3_N_4_ positive electrode, pronounced Bi^3+^/Bi^2+^ and Fe^3+^/Fe^2+^ redox couples, together with a *b*-value close to 0.5, indicate predominantly diffusion-controlled battery-type storage [110]. Thus, positive-electrode BFO can be described as undergoing reversible valence-state changes in its Bi and Fe centers, coupled with electrolyte-ion transport for charge compensation. Overall, anodic BFO is more strongly associated with cation insertion/de-insertion and vacancy-assisted ion migration under reducing polarization, whereas cathodic BFO relies predominantly on diffusion-controlled multivalent Bi/Fe redox processes under positive polarization [109,110]. Al-Maswari et al. [111] synthesized a porous BFO-embedded N-doped carbon (BFO-NC) nanocomposite through a polymeric precursor route followed by calcination at 800 °C for 6 h. The composite comprised ~15 nm BFO NPs intimately integrated with a conductive N-doped graphitic carbon matrix and exhibited a high surface area of 339 m^2^ g^−1^**.** In 1.0 M Na_2_SO_4_**,** the electrode delivered a specific capacitance of 811 F g^−1^ at 50 mV s^−1^ and 682 F g^−1^ at 30 A g^−1^**,** substantially outperforming pristine BFO. The enhanced response was attributed to the porous architecture, enlarged electroactive surface area, improved electronic transport, and more efficient SO_4_^2−^ ion diffusion through the carbon framework. The electrode also showed stable cycling over 5000 cycles, underscoring the value of carbon–perovskite integration for overcoming the intrinsic transport limitations of BFO-based supercapacitor electrodes. BFO-based nanocomposites have been widely explored for enhancing electrochemical performance through interfacial and synergistic effects. Sol–gel-derived BFO-MoS_2_ binary and BFO-MoS_2_-carbon (graphene or MWCNT) ternary nanocomposites have been successfully synthesized [112]. While carbon incorporation effectively tunes the optical band gap, electrochemical studies indicate that the binary BFO-MoS_2_ system exhibits stronger synergistic behavior than the ternary counterparts, suggesting possible interfacial competition in multicomponent architectures. A ternary BFO/MoS_2_@MWCNT nanocomposite electrode was developed for asymmetric supercapacitors. In this architecture, MoS_2_ provided abundant redox-active sites, while MWCNTs enhanced electrical conductivity and structural integrity, enabling efficient charge transfer and ion diffusion [113]. The synthesis process has been displayed in Figure 10.

**Figure 11:**
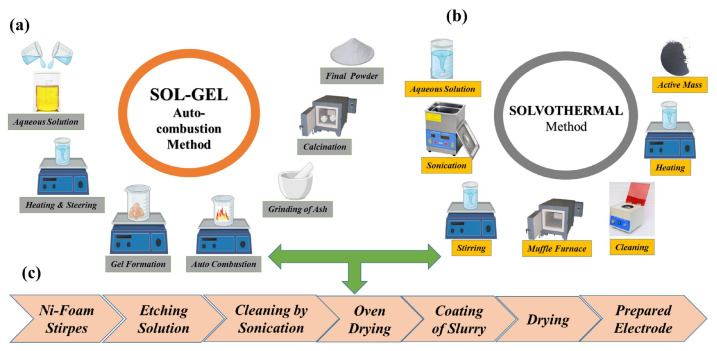
Schematic diagram shows the preparation of BFO and BFO/rGO composite using (**a**) sol–gel method, (**b**) solvothermal method and (**c**) electrode fabrication process. Reproduced with permission under license CC BY 4.0 [115].

The optimized electrode exhibits a high specific capacitance of 1765 F g^−1^ at 1 A g^−1^ with an excellent rate capability. The assembled asymmetric device delivers an energy density of 65.7 Wh kg^−1^ at a power density of 802.7 W kg^−1^ and retains 96.7% capacitance after 10,000 cycles, demonstrating superior cycling stability. In contrast, g-C_3_N_4_ functionalization of BFO significantly improves charge-storage performance, increasing specific capacitance from 557 to 1164 F g^−1^, reducing charge-transfer resistance (0.3 Ω), and maintaining excellent cycling stability over 5000 cycles [114]. The enhanced performance is attributed to increased surface area, nitrogen-rich conductive pathways, and accelerated ion transport. In another study [115], BFO/reduced graphene oxide (rGO) composites were also synthesized via sol–gel and solvothermal routes exhibiting enhanced conductivity, surface area, and redox activity (Figure 11a,b). Figure 11c shows the fabrication of BFO and BFO/rGO composite-based electrodes.

Increasing rGO content significantly improves electrochemical performance, with optimized compositions achieving specific capacitance up to 877 F g^−1^ and 90% retention over 3000 cycles. Carbon-stabilized BFO nanostructures exhibit an enhanced electrochemical activity, stability, and voltage window, enabling high-performance hybrid energy storage. Synergistic integration with layered chalcogenides and nitrogen-rich carbon frameworks establishes BFO-based hybrids as promising electrodes for high-energy, high-voltage supercapacitors.

### 3.4. BFO/MXene-Based Materials

Hierarchical BFO-MXene-based composites have emerged as multifunctional materials for energy storage and electrocatalysis. A BFO/Cr_2_CT_x_ MXene heterostructure, synthesized via selective etching of the Cr_2_AlC MAX phase, exhibited excellent bifunctional performance as a supercapacitor electrode. The composite delivered a high specific capacity of 671.2 C g^−1^ at 1 A g^−1^ with 90% retention after 3000 cycles, demonstrating excellent stability [116]. Similarly, ternary nanocomposites composed of 0D BFO NPs, 1D multi-walled carbon nanotubes, and 2D Ti_3_C_2_T_x_ MXene nanosheets exhibited strong synergistic effects, significantly enhancing electrochemical performance. Among the studied systems, the optimized composite (MBC2) achieves a high specific capacitance of 1300 F g^−1^, an energy density of 231 Wh kg^−1^, and a power density of 1.26 kW kg^−1^ within a 1 V window, underscoring the effectiveness of multidimensional conductive networks in facilitating rapid ion/electron transport [117]. The electrochemical performance of the BFO-based materials for supercapacitors has been summarized in Table 2.

It can be observed from Table 2 that the doping approach and conductive hybridization improved the supercapacitor performance of BFO-based supercapacitors. Mn-doped BFO showed the highest specific capacitance (1795 F g^−1^ at 1 A g^−1^), followed by BFO/MoS_2_@MWCNTs (1765 F g^−1^) and BFO/Cr_2_CT_x_ MXene (1513 F g^−1^). BFO@NiO showed the longest cycling test of 20,000 cycles whereas MXene-CNTs-BFO exhibited a stability of 13,000 cycles. However, direct comparison might be difficult because the reported studies used different current densities, scan rates, and testing conditions.

## 4. Conclusions and Perspectives

BFO-based materials have been widely investigated as supercapacitor electrodes, although their development remains at a relatively early stage compared with more established electrode materials. The studies reviewed here show that the electrochemical behavior of BFO cannot be explained by its nominal BFO composition alone. Its charge-storage performance is governed by several closely related factors, including phase purity, Bi/Fe stoichiometry, multivalent redox chemistry, oxygen vacancy concentration, lattice distortion, particle size, exposed crystal surfaces, electronic conductivity, electrode architecture, and electrolyte composition. Consequently, the most meaningful advances have come not from pristine BFO itself, but from the controlled modification of these structural, chemical, and interfacial features. The available synthesis methods each offer distinct advantages and limitations. Sol–gel processing provides good compositional homogeneity and considerable flexibility for dopant incorporation, but the final product is highly sensitive to precursor coordination, Bi volatilization, and thermal treatment. Hydrothermal and solvothermal methods allow better control over particle morphology, anisotropic growth, and exposed facets, although their narrow phase purity windows and strong dependence on alkalinity can affect reproducibility. Co-precipitation is simple and potentially suitable for large-scale production, but careful control of local pH, precursor speciation, and the order of reagent addition is required. Microwave-assisted synthesis can greatly reduce processing time, although the products may be highly sensitive to irradiation power, duration, and heating uniformity. Mechanochemical and solid-state routes are attractive for large-scale preparation and defect or interface engineering, but contamination, incomplete phase conversion, and the need for post-annealing remain important concerns.

Future work should therefore move beyond the empirical adjustment of particle size and calcination temperature and instead focus on mechanism-based control of phase formation, defect chemistry, and electrode-relevant microstructure. Substantial improvements in electrochemical performance have already been achieved through stoichiometric control, aliovalent doping, oxygen vacancy engineering, nano-structuring, and heterointerface formation. BFO systems modified with Mn, Sr, Zn, V, and rare-earth elements have demonstrated how substitution can influence the electronic structure, lattice strain, defect population, ion migration barriers, and accessibility of electroactive sites. Similarly, integrating BFO with conductive carbon frameworks, graphitic carbon nitride, layered chalcogenides, or MXenes can compensate for its poor intrinsic conductivity and provide more efficient pathways for electron and ion transport. Nevertheless, greater compositional complexity does not necessarily produce better performance. Excessive doping can promote secondary-phase formation, while multicomponent composites may contain poorly connected active material, competing interfaces, or electrochemically inaccessible mass. Such complexity can also make the actual charge-storage mechanism difficult to identify. The next stage of BFO research should therefore establish measurable relationships among structure, defects, interfaces, and electrochemical performance rather than continuing to add components without clear mechanistic justification. This is particularly important given the wide variation in reported performance among doped BFO electrodes, BFO/carbon and BFO/MXene composites, and high-voltage hybrid devices. The charge-storage behavior of BFO is also more complex than the general description of a “pseudocapacitive oxide” suggests. Depending on the electrode polarity, defect state, electrolyte, and operating potential window, BFO may exhibit surface or near-surface Faradaic reactions, mixed capacitive and diffusion-controlled behavior, reversible Li^+^ insertion and extraction, oxygen vacancy-assisted ion transport, and redox processes involving both Bi and Fe centers. In oxygen-deficient BFO, reversible structural changes have been associated with Li^+^ intercalation and de-intercalation within the BFO lattice, where oxygen vacancies and local electric fields can facilitate ion migration. Although this mechanistic diversity is promising, it also highlights a major unresolved problem. Similar electrochemical profiles are often assigned to different charge-storage processes without sufficient experimental evidence. Future studies must clearly distinguish intercalation pseudocapacitance from surface redox reactions, battery-type diffusion-controlled storage, irreversible conversion reactions, electrolyte decomposition, and electrochemical contributions from secondary phases. This will require a greater use of complementary kinetic analysis, operando or in situ characterization, post-cycling structural examination, and appropriate control electrodes. Another important direction is the shift from optimizing individual electrodes to engineering the complete device. The pH-decoupled AC//BFO system discussed in this review shows that the local electrolyte environment, ion-selective membrane, membrane resistance, and suppression of water decomposition can be jointly controlled to extend the usable voltage window of aqueous devices and improve their energy density. This strategy is important because energy density cannot be increased indefinitely by raising specific capacitance alone. The operating voltage, electrode mass balance, electrolyte stability, ion transport, and separator design are equally important. However, the capacitance loss and self-discharge reported for such configurations indicate that several practical challenges remain. Ion crossover, membrane resistance, parasitic reactions, electrolyte imbalance, and long-term degradation of electrode–electrolyte interfaces must be addressed before these systems can reach technological maturity. Overall, future progress will depend on combining controlled BFO chemistry with reliable mechanistic evidence and carefully designed full-cell architectures.

## Figures and Tables

**Table 1:** Comparative synthesis conditions for BFO-based materials prepared using different methods.

Synthetic Method	Materials	Temperature (°C)	Calcination (°C)/Time	Reaction Time	Crystallite Size (nm)	Ref.
Sol–gel	BFO	80	300 (1 h)	Stirring until complete dissolution, followed by approximately 48 h at 80 °C	24	[42]
Sol–gel	BFO	80	500 (1 h)	Stirring until complete dissolution, followed by approximately 48 h at 80 °C	33	[42]
Sol–gel	BFO	80	700 (1 h)	Stirring until complete dissolution, followed by approximately 48 h at 80 °C	82	[42]
Sol–gel	BFO	80	200	120 min stirring of each precursor solution, followed by 8 h heating after mixing	16.11	[43]
Sol–gel	BFO	80	200 (1 h)	-	25	[44]
Sol–gel	BFO	70	450 (4 h)	-	9.1	[45]
Hydrothermal	BFO	175 (6 h)	-	-	-	[58]
Hydrothermal	BFO	180 (24 h)	550 (2 h)	-	23.2	[59]
Hydrothermal	BFO	140 (72 h)	-	-	23.92	[65]
Sonochemical	Bi_0.98_Sm_0.02_FeO_3_ nanorods	-	525 (2 h)	105 min (sonication time)	65	[75]
Co-precipitation	BFO	-	500 (1 h)	-	31	[76]
Microwave-assisted method	BFO/Bi_2_O_3_/Fe_2_O_3_	-	500 (24 h)	30 min microwave irradiation	35	[84]
Microwave synthesis	BFO	180	500 (1 h)	30 min microwave reaction	-	[86]
Microwave-assisted sol–gel synthesis	BFO-Bi_25_FeO_40_ film	-	500 (0.5/1 h)	-	19.85	[88]
Solid-state synthesis	BFO	-	750 (1 h)	-	48	[89]
Mechano-thermal synthesis	BFO-Fe_2_O_3_	-	750 (1 h)	-	-	[91]
Direct mechanochemical	BFO	-	-	-	-	[94]

**Table 2:** Electrochemical performance of BFO-based electrode materials for supercapacitors.

Materials	Specific Capacitance (F g^−1^)	Current Density (A g^−1^)/Scan Rate	Cyclic Stability	Ref.
BFO@NiO	772	1 mV/s	20,000	[17]
BF-xCT	86.4	10 mV/s	1150	[18]
Nd-BFO/g-C_3_N_4_@Ppy	645	8 mA cm^−2^	5000	[19]
Bi_1+x_FeO_3_	222.2	2 mV/s	10,000	[30]
Nd-BFO	24	2	2000	[31]
BFO/MoS_2_@MWCNTs	1765	1	10,000	[34]
Mn-BFO	1795	1	5000	[99]
AC//BFO (CEM-2)	61	1	5000	[100]
Sr-doped BFO	1200	1	5000	[101]
Zn-doped BFO	223	0.2	10,000	[102]
PVDF-β-Bi_2_O_3_@BFO	300	0.5	10,000	[105]
BF/BT-doped graphene polymer structure	721.8	1	-	[108]
N/S/P-CNSF//GF@BFO-NC BSH	294	1	10,000	[109]
BFO/g-C_3_N_4_	330	1	-	[110]
BFO	811	5 mV/s	-	[111]
BFO-MoS_2_	244.74	5 mV/s	-	[112]
BFO-MoS_2_-MWCNTs	208.43	5 mV/s	-	[112]
BFO-MoS_2_-graphene	157.86	5 mV/s	-	[112]
BFO/MoS_2_@MWCNTs	1765	1	10,000	[113]
BFO@g-CN	1164	1	5000	[114]
BFO–rGO	877	1.95	3000	[115]
BFO/Cr_2_CT_x_ MXene	1513	1	3000	[116]
MXene-CNTs-BFO	942.8	1	13,000	[117]

## Data Availability

No new data were created or analyzed in this study. Data sharing is not applicable to this article.
